# Dry Powder Inhalers for Proteins Using Cryo-Milled Electrospun Polyvinyl Alcohol Nanofiber Mats

**DOI:** 10.3390/molecules27165158

**Published:** 2022-08-12

**Authors:** Takaaki Ito, Eriko Yamazoe, Kohei Tahara

**Affiliations:** Laboratory of Pharmaceutical Engineering, Gifu Pharmaceutical University, 1-25-4 Daigaku-Nishi, Gifu 501-1196, Japan

**Keywords:** protein formulation, protein delivery, stability, inhalation, pulmonary drug delivery, milling, powder technology, biodegradable polymer, polymeric drug carrier, porosity

## Abstract

To enable the efficient delivery of drugs to the lungs, the drug particle design for most dry powder inhalers (DPIs) involves reducing the aerodynamic particle size to a few microns using methods such as spray-drying or jet-milling. Stresses, including heat and the shear forces generated by the preparation processes, may result in the degradation and denaturation of drugs such as those based on peptides and proteins. Here, we showed that cryo-milled polyvinyl alcohol nanofiber mats loaded with α-chymotrypsin by electrospinning exhibited suitable inhalation properties for use in DPIs, while maintaining enzymatic activity. The cryo-milled nanofiber mats were porous to fine particles, and the particle size and drug stability depended on the freezing and milling times. The median diameter of the milled fiber mats was 12.6 μm, whereas the mass median aerodynamic diameter was 5.9 μm. The milled nanofiber mats were successfully prepared, while retaining the enzymatic activity of α-chymotrypsin; furthermore, the activity of milled fiber mats that had been stored for 6 months was comparable to the activity of those that were freshly prepared. This novel method may be suitable for the DPI preparation of various drugs because it avoids the heating step during the DPI preparation process.

## 1. Introduction

The global sales of biopharmaceuticals, including protein-based therapeutics, have grown rapidly, and drugs based on these compounds made up the majority of the top 100 drugs sold in 2019 [1]. The advantages of biopharmaceuticals include their target-specific action and flexible pharmacological design; thus, their market share will likely increase in the future. Conversely, these products have room for improvement regarding pharmaceutical formulation design. For instance, although biopharmaceuticals are generally administered in solution via injection, these products have strict storage requirements and raise concerns about invasiveness [2]. In light of these issues, formulation technologies are being developed to improve storage stability and establish alternative administration routes.

In the case of respiratory epithelial diseases, the development of biopharmaceutical products such as inhalers is a rational vehicle for their drug delivery [3,4]. Inhalation drug products are less invasive than injections and can reduce systemic side effects. Moreover, the biopharmaceutical inhalations can be self-administered by patients and may increase compliance of patients. The types of devices include inhalation solutions (soft mist inhaler and nebulizer), pressurized metered-dose inhalers, and dry powder inhalers (DPIs), which are selected based on patient needs, pathology, and the physicochemical properties of the active pharmaceutical ingredient.

However, most inhalation drug products are subjected to physicochemical stresses such as hydrolysis, ultrasound, shear forces, and heating at some point during the process spanning from manufacture to administration. These stresses can cause the degradation and denaturation of peptides and proteins, and have hampered the clinical application of inhaled biopharmaceuticals.

To solve these problems, we became interested in the preparation of inhalation biopharmaceutical DPIs using electrospinning techniques. DPIs are a promising device for the delivery of biopharmaceuticals, which are labile in solution and undergo hydrolysis during storage. Electrospinning is an electrospray ionization technique that prepares nanofiber mats when the polymer solutions are released by overcoming their surface tension via the application of an electrical force [5,6]. Polymer solvents are evaporated as they travel toward the collector without the use of shear forces and heating.

To prepare DPIs, an aerodynamic diameter, calculated from the geometric diameter and density, should be achieved in the range of 1–6 μm [7,8]. In general, the drugs are milled to single-micron-sized particles by jet-milling and spray-drying [9]. However, single-micron-sized particles have high adhesion, which leads to their aggregation into large particles that interfere with dispersion from inhalers [10,11]. To prevent aggregation, the particles must be mixed with large carriers (50–100 μm) such as lactose [12]. On the other hand, these mixtures require strong inspiration to separate the drug from the carrier, which raises concern about the influence of these carriers on inhalation characteristics, depending on the inhalation technique of the patient. Furthermore, several stresses including shear force and heating generated by jet-milling and spray-drying can cause the degradation and denaturation of biopharmaceuticals [8,13]. The appropriate processing of polymeric nanofiber mats with a large specific surface area and high porosity has the potential to prepare low-density particles with excellent inhalation properties for DPIs.

Thus, in this study, we aimed to develop therapeutic protein-based drugs as DPIs by milling electrospun nanofiber mats. We prepared nanofiber mats composed of α-chymotrypsin (α-Chy) and polyvinyl alcohol (PVA) as the experimental drug and excipient, respectively. α-Chy is a digestive enzyme, and the α-Chy activation method is often used to measure enzyme activity in vitro [14]. α-Chy is inactivated by heating or in solution and must be frozen for long-term storage [15]. PVA is a biodegradable synthetic polymer, which is approved as a Japanese pharmaceutical excipient. In our previous study, we reported on preparing drug-loaded PVA nanofibers as a solid dispersion system using electrospinning technique [6]. The milling powders were prepared using a cryo-mill and evaluated in terms of in vitro aerosol performance and enzyme activity.

## 2. Results

### 2.1. Physicochemical Properties of Electrospun Nanofiber Mats and Milled Nanofiber Mats

**Figure 1:**
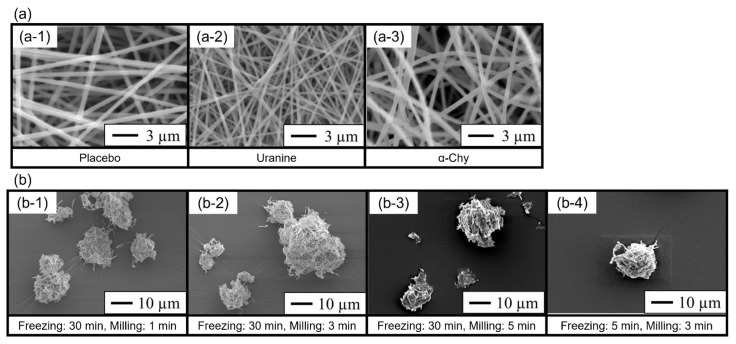
Scanning electron micrographs of the electrospun nanofiber mats and milled nanofiber mats. (**a**) Nanofiber mats prepared using the electrospinning technique. (**a-1**) A polyvinyl alcohol (PVA) nanofiber mat without the experimental drugs, (**a-2**) the PVA nanofiber mat loaded with uranine, and (**a-3**) the PVA nanofiber mat loaded with α-chymotrypsin (α-Chy). (**b**) Uranine-loaded PVA nanofiber mats milled by cryo-milling. (**b-1**) freezing: 30 min, milling: 1 min; (**b-2**) freezing: 30 min, milling: 3 min; (**b-3**) freezing: 30 min, milling: 5 min; and (**b-4**) freezing: 5 min, milling: 3 min.

We prepared electrospun nanofibers from PVA solutions containing α-Chy or uranine. Most of the electrospun nanofibers observed using a scanning electron microscope were 300–600 nm in diameter (Figure 1a). The milled nanofiber mats were prepared for use in DPIs by cryo-milling (Figure 1b). Most of the milled nanofiber mats had a geometric diameter of approximately 5–30 μm. The milled nanofiber mats that had been frozen for 30 min and milled for 1 and 3 min were powdered with porous particles, while maintaining the fiber structure. Conversely, the milling of the nanofiber mat powder for 5 min destroyed its fiber structure. In addition, the short freezing time (5 min) also affected the particle shape and destroyed the fiber structure.

We calculated the geometric diameter of the milled fiber mats using a dry laser diffraction particle size analyzer (Table 1). D_10_, D_50_, D_90_, and Span were determined from the cumulative particle size distribution. Among the nanofiber mats that were milled for 3 min, the freezing time (5 and 30 min) did not affect the geometric diameter (14.37 and 12.60 μm, respectively). In contrast, the geometric diameter decreased inversely to the milling time, and the median diameter (D_50_) of the fiber mats that were milled for 5 min was the smallest, with a value of 7.36 μm.

### 2.2. In Vitro Aerosol Performance of the Milled Nanofiber Mats

**Figure 2:**
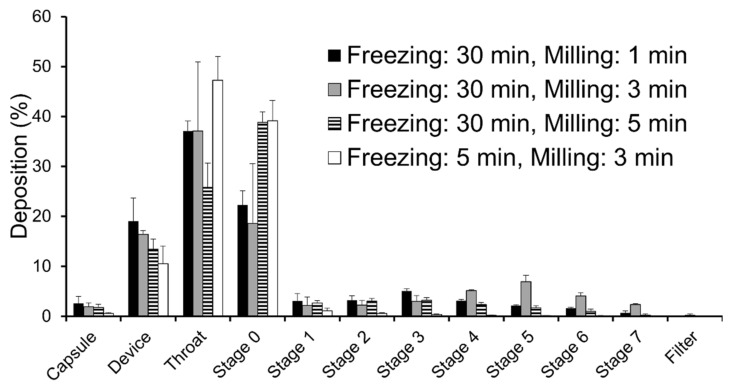
Deposition patterns of the milled nanofiber mats in an eight-stage Andersen cascade impactor. Freezing: 30 min, milling: 1 min (black); freezing: 30 min, milling: 3 min (gray); freezing: 30 min, milling: 5 min (stripe); and freezing: 5 min, milling: 3 min (white). Each value represents the mean ± standard deviation (SD; n = 3).

Three milligrams of each milled nanofiber mat as the total payload were aspirated to ACI (Figure 2). The DD values corresponding to inhaled drug volumes for all milled nanofiber mats were over 75%, which increased as milling time increased for the same freezing time (Table 2). Conversely, the FPF and MMAD varied significantly according to the freezing and milling conditions, with the nanofiber mat that was frozen for 30 min and milled for 3 min exhibiting the highest aerosol performance. This milled nanofiber mat showed a maximum FPF of 26.5%, which was 20-fold higher than that of the nanofiber mat that was frozen for 5 min and milled for 3 min. For the same freezing time, the nanofiber mats that were milled for less or longer than 3 min exhibited a decreased aerosol performance.

### 2.3. Enzyme Activities of Milled Nanofiber Mats Containing α-Chymotrypsin

**Figure 3:**
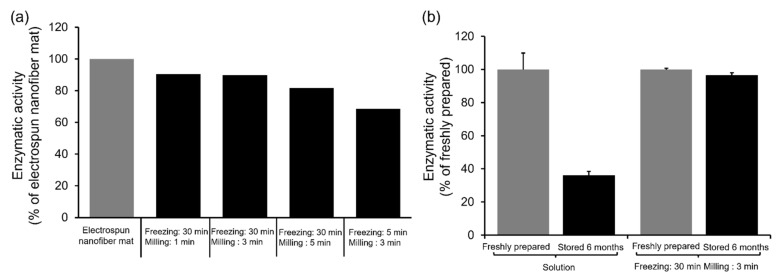
Assessment of the enzymatic activity of α-chymotrypsin (α-Chy) via the determination of p-nitrophenol amounts. (**a**) Enzymatic activity of α-Chy-loaded polyvinyl alcohol (PVA) nanofiber mats milled by cryo-milling (*n* = 1). The vertical axis indicates the activity of the α-Chy-loaded PVA nanofiber mat. (**b**) Long-term stability of α-Chy (*n* = 3, mean ± standard deviation [SD]). The milled nanofiber mats that were frozen for 30 min and milled for 3 min and solutions containing α-Chy of the same composition were stored for 6 months and compared with freshly prepared ones regarding enzymatic activity.

The enzymatic activities of milled nanofiber mats containing α-Chy were measured to estimate the integrity of the protein after milling. As a result, the electrospinning–cryo-milling process retained the enzymatic activity in the nanofiber mats, although it was partially destroyed depending on milling time (Figure 3a). The enzyme activities of all milled nanofiber mats frozen for 30 min were over 80%, and decreased according to the milling time. Conversely, the enzymatic activity of nanofiber mats frozen for 5 min and milled for 3 min was lower than that of the mats that were frozen for 30 min (68.6%).

To confirm the long-term stability of α-Chy, the milled nanofiber mats that were frozen for 30 min and milled for 3 min, and solutions containing α-Chy of the same composition were stored for 6 months (Figure 3b). The activity of the milled nanofiber mats that were stored for 6 months was comparable (96.4%) to that of the mats that were freshly prepared. Conversely, the α-Chy solution, which was stored as a control, showed a decrease in enzymatic activity of 36.0% after 6 months of storage.

## 3. Discussion

In this study, we aimed to develop therapeutic protein-based drugs as DPIs using electrospun nanofiber mats. The electrospinning technique should be useful for the preparation of heat-sensitive drugs to be used in DPIs because it does not require heating. Moreover, the milled nanofiber mats are a potential solution to supply pharmaceutics conveniently and economically for drugs that are labile in solution and undergo hydrolysis during storage.

During particle design for DPIs, it is important that the aerodynamic diameter remain in the range of 1–6 μm [7,8]. Conversely, single-micron-sized particles have high adhesion, which interferes with dispersion from inhalers [10,11]. Regarding this point, the milled nanofiber mats prepared in this study retained their fiber structure and had low-density porous shapes, with a geometric diameter of approximately 5–30 μm; thus, they would be expected to exhibit low adhesion and easy dispersion [16]. Because the aerodynamic diameter is proportional to the square root of its density, the porous structures have an aerosol performance that is superior to the apparent particle size.

In the present study, the electrospinning–cryo-milling process greatly affected the geometric diameter and the aerosol performance. The results presented in Figure 2 and Table 2 show that the milled nanofiber mats that were frozen for 30 min had a more favorable aerosol performance than those that were frozen for 5 min, despite the application of an identical milling time. Although the two milled nanofiber mats had comparable geometric diameters (Table 1; 12.60 μm and 14.37 μm, respectively), the fiber structure of the milled nanofiber mats that were frozen for 5 min was destroyed (Figure 1(b-4)). This prompts two suggestions: that the low-density porous shape is useful for achieving a favorable aerosol performance, and that freezing for 5 min is insufficient to reduce the particle size and maintain the structure. According to Fourier’s law and Newton’s cooling law, the fiber structure might prolong the time needed for freezing because of the low-heat convection in the sample cell of the cryo-mill and the low-heat conduction attributable to the small cross-sectional area [17]. We consider that prolonged freezing can prevent aggregation through folding by decreasing the flexibility of the fibers. Conversely, for the same freezing time, the nanofiber mats that were milled for 3 min had the highest aerosol performance. Moreover, the geometric diameter decreased in inverse proportion to the milling time (Table 1), and the milled nanofiber mats that were milled for 1 or 3 min retained their fiber structure, whereas those that were milled for 5 min disappeared (Figure 1b). These results suggest that very short milling times are insufficient for producing fine particles, whereas excessive milling times destroy the fiber structure and increase the apparent density of the milled nanofiber mats. In future investigations, protein-loading particles for DPIs with better aerosol performance should be prepared by optimizing the nanofiber materials and manufacturing processes.

As shown in Figure 3, we successfully prepared the milled nanofiber mats, while retaining the enzymatic activity, although a partial reduction in activity was observed. The observed deactivation of α-Chy depending on the milling time should be improved in future studies. The milled nanofiber mats that were frozen for 30 min exhibited a more favorable enzymatic activity than those that were frozen for 5 min (Figure 3a). The significant decomposition of the powder that was frozen for 5 min was probably caused by insufficient freezing. Moreover, DPIs containing α-Chy showed a superior storage stability compared with storage in solution (Figure 3b).

In the present study, we proposed the electrospinning–cryo-milling process as an alternative method for the preparation of DPIs. Conversely, the design space should be identified in future studies because the aerosol performance, the uniformity of geometry distribution, and drug integrity of the DPIs prepared using this process are affected by various factors such as formulation, the electrospinning process, and milling conditions [18,19]. The enzyme activity of the PVA nanofiber mats after electrospinning was about 12% lower than that of untreated α-Chy solution. Therefore, the optimization of the electrospinning process will also be investigated to improve the yield. For instance, clarifying the relationships between the enzymatic activity and physicochemical stresses applied during the electrospinning process, such as voltage and nozzle shear, can be useful information for optimizing the process [20]. Otherwise, protection of the nanofibers by the core-shell structure may improve the yields of the protein [14]. Other polymers that have been employed as fiber excipients include poly (l-lactide) and poly (*ε*-caprolactone) [21,22]. In this study, we found that cryo-milling was useful as a powdering method for electrospun fibers. Thieme et al. milled electrospun fibers in liquid nitrogen using a motor-driven blade [23]. Reducing the fiber milling process would directly lead to improvements in drug stability. Furthermore, the electrospray technique, which is another electrospray ionization technique, might be a promising DPI preparation method because it can prepare single-micron-sized particles without shear forces and heating [19].

In the rapidly expanding market for biopharmaceuticals, inhalation therapies that achieve a noninvasive administration are a useful application. The establishment of this novel pharmaceutical technology for inhalation therapies offers flexibility for drug development.

## 4. Materials and Methods

### 4.1. Preparation of α-Chy-Loaded Electrospun PVA Nanofiber Mats

PVA (GOHSENOL EG-40P; degree of polymerization, 2400; degree of hydrolysis, 88 mol%) was provided from Mitsubishi Chemical Co. (Tokyo, Japan) and used as the fiber excipient [6]. α-Chy was purchased from Sigma-Aldrich (St. Louis, MO, USA) and used as the experimental drug. Uranine was purchased from Tokyo Chemical Industry Co., Ltd. (Tokyo, Japan) and used as a fluorescent label of dry powders for in vitro aerosol performance.

PVA was dissolved in distilled water at 80 °C with stirring, and subsequently brought to room temperature. The sample solution was prepared by adding the drug (α-Chy or uranine) to the PVA solution. The total solute concentration was adjusted to 320.0 mg/4.0 mL. The composition ratio of the drug was set to 1% (3.2 mg) to minimize the effect of the physicochemical properties of electrospun nanofiber mats.

For the electrospinning process, the sample solution was loaded into 5-mL syringes and fed onto 22-gauge needles using a syringe pump (Yutaka Electric Co., Gifu, Japan) at 0.5 mL/h. The electrode of the high-voltage power supply (MECC CO., LTD., Fukuoka, Japan) was clamped to the needle, and the aluminum-covered collector was grounded as a cathode. The remaining electrospinning conditions used here were as follows: the distance between the needle and the collector was 10 cm; the applied voltage was 10 kV; and the experiments were performed at room temperature (20–25 °C) at a relative humidity below 50%.

### 4.2. Electrospinning/Cryo-Milling Processes of Electrospun Nanofiber Mats

The milled powders were prepared using a cryo-mill (JFC-300, Japan Analytical Industry Co., Ltd., Tokyo, Japan). The electrospun nanofiber mats (approximately 50 mg) were placed in a sample cell (12 mL) containing two steel balls with diameters of 15 mm. The sample cell was immersed in liquid nitrogen for different amounts of time (5 or 30 min), and then milled for different amounts of time (1, 3, or 5 min).

### 4.3. Physicochemical Properties of Electrospun Nanofiber Mats and Milled Nanofiber Mats

A scanning electron microscope (JSM-6510LV, JEOL Ltd., Tokyo, Japan) captured the morphology of the electrospun nanofiber mats and milled nanofiber mats using an accelerating voltage at 5 kV. Prior to observation, the samples were sputtered with platinum using an auto fine coater (JFC-1600, JEOL Ltd.).

A dry laser diffraction particle size analyzer (LDSA-SPR 3500A, MicrotracBEL Corp., Osaka, Japan) equipped with a dry dispersing apparatus was used to measure the particle size distribution of the milled nanofiber mats. Three geometric diameter values (D_10_, D_50_ (median), and D_90_) were determined from the cumulative particle size distribution. Moreover, as the distribution width of the measured particle size distribution, the volume-based size distribution (Span) was calculated from the formula (D_90_ − D_10_)/D_50_.

### 4.4. In Vitro Aerosol Performance

The aerosol performance of milled nanofiber mats containing uranine were calculated using an eight-stage Andersen cascade impactor (ACI; AN-200, Tokyo Dylec Corp., Tokyo, Japan), which is listed in the Japanese Pharmacopoeia as a tool for DPI aerosol performance evaluation. Prior to inspiration, stainless-steel collection plates were coated with glycerin to prevent powder bounce. In total, 3 mg of each milled nanofiber mat were loaded onto No. 2 hydroxypropyl methylcellulose hard capsules (Qualicaps Co., Ltd., Nara, Japan), and were set in a DPI (Jethaler^®^ reverse type, Tokico System Solutions, Ltd., Kanagawa, Japan) (depressure drop, 8.7 kPa; at a flow rate of 28.3 L/min). The inhaler was then fixed to the ACI, and the flow rate and inspiration time were set to 28.3 L/min and 5 s, respectively. After inspiration, the powder deposited on each stage was rinsed with 10 mL of phosphate-buffered saline. The deposited powder amounts were estimated from the fluorescence intensity of uranine, which was measured using a GloMax-Multi Detection System (Promega Co., Madison, WI, USA) (Ex, 490 nm; Em, 510–570 nm). The aerosol performance was evaluated by the delivered dose (DD, Equation (1)), fine particle fraction (FPF, Equation (2)), and mass median aerodynamic diameter (MMAD), as calculated from the percentage of powder deposition at each stage [24]. In turn, the DD is an indicator of the output efficiency from the inhaler and the capsule. FPF is an indicator that estimates the accessibility of the powder into the lungs (aerodynamic diameter <4.7 μm). Finally, the MMAD is determined by plotting the cumulative percentage of deposition patterns on a logarithmic normal probability paper.

DD (%) = (mass in throat and lower parts)/(total mass) × 100(1)

FPF (%) = (mass in stage 3 and lower parts)/(mass in throat and lower parts) × 100(2)

### 4.5. Enzymatic Activities of Milled Nanofiber Mats Containing α-Chymotrypsin

The enzymatic activities of α-Chy were determined based on the amount of *p*-nitrophenyl acetate de-esterification to *p*-nitrophenol [14]. We dissolved 22.5 mg of the milled nanofiber mats in 1.5 mL of phosphate-buffered saline to an α-Chy concentration of 150 μg/mL for 1 h. Subsequently, 20 μL/60 mM *p*-nitrophenyl acetate in dimethyl sulfoxide (FUJIFILM Wako Pure Chemical Co., Osaka, Japan) were added to the dissolved solutions and reacted for 30 min. The absorption of the reacted solutions was measured at 404 nm using a UV–Visible spectrophotometer (UV-1800, Shimadzu, Kyoto, Japan) to determine the amount of *p*-nitrophenol. The enzymatic activities of α-Chy in the milled nanofiber mats were calculated in comparison with those detected in the nanofiber mats.

In addition, to confirm their long-term stability, we stored the milled nanofiber mats containing α-Chy for 6 months. Approximately 25 mg of milled nanofiber mats were placed in glass vials, which were covered with fabric and secured with rubber bands. The vials were stored in airtight containers with silica gel at 20–25 °C. We also stored a freshly prepared solution including α-Chy with the same composition as that of the DPI, as a control.

### 4.6. Statistical Analysis

The statistical analyses of the aerosol performance by ACI were carried out using Tukey’s test (JMP 15 software, SAS Institute Inc., Cary, NC, USA).

## 5. Conclusions

In this study, nanofiber mats milled using a cryo-mill showed excellent aerosol performance and drug integrity. We found that milled nanofiber mats for inhalation could be created by optimizing the milling conditions. Long freezing times were necessary for the electrospun nanofibers, whereas long milling times caused aggregation and drug degradation. This novel preparation technique is available for the preparation of DPIs for various drugs, including those based on therapeutic proteins, because heating is not essential. These results offer novel preparation methods for the formulation of DPIs.

## Figures and Tables

**Table 1:** Operating Conditions of Freeze Milling and Particle Size of the Milled Nanofiber Mats.

Formulation	D_10_ (µm)	D_50_ (µm)	D_90_ (µm)	Span
Freezing: 30 min, milling: 1 min	8.73 ± 1.72	34.39 ± 1.22	54.99 ± 2.45	1.35 ± 0.10
Freezing: 30 min, milling: 3 min	6.91 ± 0.67	12.60 ± 0.67	24.25 ± 3.04	1.38 ± 0.26
Freezing: 30 min, milling: 5 min	3.90 ± 0.27	7.36 ± 0.80	15.11 ± 6.33	1.48 ± 0.68
Freezing: 5 min, milling: 3 min	4.89 ± 2.83	14.37 ± 1.71	28.83 ± 1.49	1.69 ± 0.33

A dry laser diffraction particle size analyzer determined the geometric diameter of the milled nanofiber mats (mean ± standard deviation [SD], *n* = 3).

**Table 2:** Aerosol performance of the milled nanofiber mats.

Formulation	DD (%)	FPF (%) **^b^	MMAD (µm)
Freezing: 30 min, milling: 1 min	78.4 ± 3.6 *^a^	16.3 ± 1.3	9.7 ± 1.0
Freezing: 30 min, milling: 3 min	81.8 ± 0.4	26.5 ± 0.8	5.9 ± 3.4
Freezing: 30 min, milling: 5 min	83.8 ± 2.8	10.7 ± 1.2	11.0 <
Freezing: 5 min, milling: 3 min	88.9 ± 3.9	0.9 ± 0.3	11.0 <

DD, delivered dose; FPF, fine particle fraction; MMAD, mass median aerodynamic diameter (mean ± standard deviation [SD], *n* = 3). The asterisks indicate significant differences, as analyzed by Tukey’s test. *a: *p* < 0.05 (compared with freezing for 5 min and milling for 3 min), **b: *p* < 0.01 (significant differences between all groups).

## Data Availability

Not applicable.

## Sample Availability

Samples of the compounds are not available from the authors.

## Abbreviations

α-Chy	α-chymotrypsin
ACI	Andersen cascade impactor
DD	delivered dose
DPI	dry powder inhaler
FPF	fine particle fraction
MMAD	mass median aerodynamic diameter
PVA	polyvinyl alcohol
